# Application of a Target-Guided Data Processing Approach in Saturated Peak Correction of GC×GC Analysis

**DOI:** 10.1021/acs.analchem.1c02719

**Published:** 2022-01-20

**Authors:** Penghan Zhang, Silvia Carlin, Pietro Franceschi, Fulvio Mattivi, Urska Vrhovsek

**Affiliations:** † Research and Innovation Center, Edmund Mach Foundation, Via E. Mach 1, San Michele all’Adige 38098, Italy; ‡ Department of Cellular Computational and Integrative Biology (CIBIO), University of Trento, Via Sommarive 9, Povo, Trento 38123, Italy

## Abstract

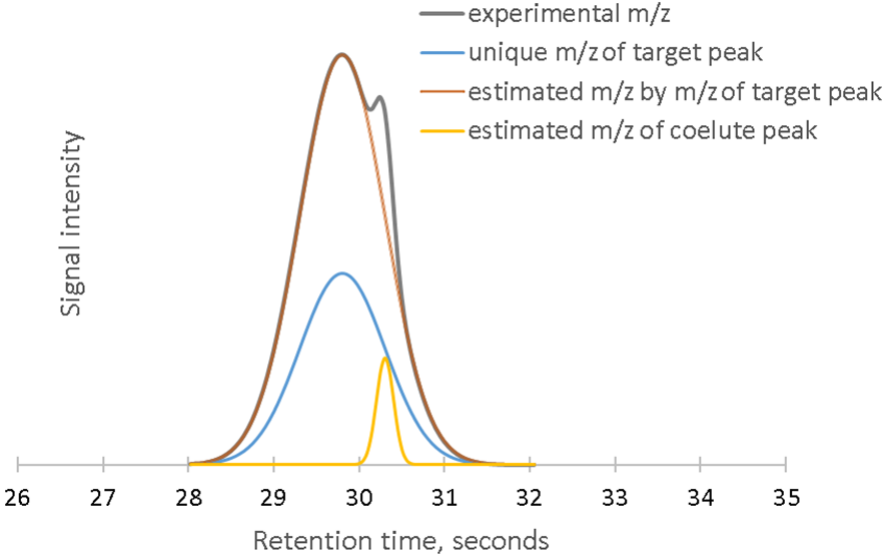

Detector and column saturations are problematic in comprehensive two-dimensional gas chromatography (GC×GC) data analysis. This limits the application of GC×GC in metabolomics research. To address the problems caused by detector and column saturations, we propose a two-stage data processing strategy that will incorporate a targeted data processing and cleaning approach upstream of the “standard” untargeted analysis. By using the retention time and mass spectrometry (MS) data stored in a library, the annotation and quantification of the targeted saturated peaks have been significantly improved. After subtracting the nonperfected signals caused by saturation, peaks of coelutes can be annotated more accurately. Our research shows that the target-guided method has broad application prospects in the data analysis of GC×GC chromatograms of complex samples.

Metabolomics technologies and the data generated by them result in a better understanding of the metabolism of many biological systems. It helps reveal the biological linkage between the genetic sequence and physiological characterization. Consequently, the biological outcome can be controlled with higher accuracy and reproducibility. The metabolomic approach has been widely applied in many fields and demonstrated successful results: microbes, food quality and crop production, animal health, and human health.^1−4^

Compared to other analytical tools, such as nuclear magnetic resonance (NMR), chromatography (gas- or liquid-based) coupled with mass spectrometry (MS) has the best overall performance in terms of efficiency, selectivity, sensitivity, and reliability.^5^ The advent of fast gas chromatography (GC)–MS has also provided the possibility to combine robust separation with a very short run time. Hence, it is widely used in metabolomics,^6^ and it is the gold standard for the analysis of volatile organic compounds. Also, liquid chromatography (LC)–MS is a very popular alternative for the broader range of compounds that can be analyzed, including the unstable compounds, with minimal sample preparation and often in a shorter run time. GC and LC have their own advantages and disadvantages in metabolomics. However, they share a similar data structure in terms of chromatograms. The chromatogram of a monodimensional chromatograph of a complex biosample is usually overcrowded. Also, an analytical method that can provide higher resolution and more separation is usually desirable. A two-dimensional (2D) chromatograph provides much increased separation capacity, chemical selectivity, and sensitivity for the analysis of metabolites present in complex samples. In a typical GC case, by adding one more dimension, the peak capacity is typically increased by 30 times.^7^ Welthagen demonstrated the power of GC×GC in the profiling of mouse spleen tissue metabolites. The GC×GC analysis identified almost 3 times as many metabolites as that identified by one-dimensional GC.^8^ The increased detection potential by 2D chromatography is extremely promising, but it also implies an additional layer of complexity in terms of data processing, in particular when relying on automatic approaches.

The complete data processing pipeline is similar to the one typically found in MS-based metabolomics and includes noise cleaning, peak picking, peak alignment, and annotation. Among the previous steps, peak picking is the most crucial one, and it becomes more challenging in the presence of 2D separation, in particular, considering that the huge differences in the concentration of the analytes (up to 8 orders of magnitude^9^) will result in detector and column saturations for the major compounds. In the case of targeted analysis, saturation phenomena could be controlled either by adjusting the column parameters or by manually tuning the detector gain. However, in a semi-quantitative untargeted study, the objective is to increase the overall analytical coverage, so coupling a higher sample concentration and an enhanced detector sensitivity with a data analysis strategy able to cope with saturation phenomena could be highly beneficial.

In the field of GC×GC, many software products are available. The best known commercial solutions are LECO’s ChromaTOF, ZOEX GC-Image, and Sepsolve ChromSpace. The above software products are discussed along with some other lesser known packages in a recent review article.^9^ Even though many software products exist, only a few algorithms for GC×GC–MS are available in journals: matched filtering, local maximum followed by parallel factor analysis (PARAFAC) unmixing, and continuous wavelet transform.^10−12^ However, when saturation occurs, these peak-picking strategies show their limitations, producing false peak splitting, which results in incorrect deconvolution, and, finally, in incorrect annotation and quantification.^13^ PARAFAC, which is recognized as the most suitable analysis method for the deconvolution of GC×GC–MS data in the field, is taken as an example.^14^ Errors may occur at the second step of PARAFAC approach, peak apex locating, due to saturation.^11^

Considering that for a biosample, the contents of major and minor compounds may vary by 9 orders of magnitude, concentration techniques are generally applied to allow the detection of trace compounds in a reliable way.^15^ Unfortunately, concentration techniques do not selectively act on minor compounds. If the minor compounds are well-sampled, major compounds are overconcentrated, leading to column/detector saturation. The experimental workaround to this problem is to perform dilutions and measure major and trace compounds at different dilution levels, but this choice turns out to be impractical for technical and economic reasons in the case of large-scale investigations (three to five technical replications would be required for each sample). Besides, peak alignment between saturated and diluted measurement could be difficult because of the retention time shift. The core idea of our paper is that, fortunately, for each metabolomic analysis, the major compounds that mainly induce the column and/or detector saturation are often known because the major components of the biosample are almost invariably known. It would then be possible to propose a “targeted” optimization of the data processing to minimize the impact of column saturation on the quantification of this targeted list of the most relevant compounds. To address the problems caused by detector and column saturation, we propose a two-stage data processing strategy that will incorporate a targeted data processing and cleaning approach upstream of the “standard” untargeted analysis. To the best of our knowledge, even if many untargeted approaches to analyze GC×GC experiments have been proposed,^16^ all of them have focused on the improvement and refinement of a pure untargeted approach, which does not take into account the saturation effect. In our proposal, with a predefined library, the annotation of saturation peaks can be achieved more robustly and accurately. Then, signals of saturated peaks are subtracted from the chromatogram. Finally, the remaining unannotated signal in the chromatogram can be processed with a general untargeted approach.

## Experimental Section

### Samples and Reagents

To benchmark the outcome of the targeted analysis and data subtraction approach, a dilution series of standard solutions (2, 20, 200, and 2000 mg/L) were prepared. The column saturation was simulated with a concentrated standard solution (200 and 2000 mg/L). According to our experience, annotations for esters are vulnerable to saturations. Hence, the prepared standard solution consisted of five esters, from apolar to polar, according to their retention time indices (RTIs): *cis*-3-hexenyl acetate (RTI = 1311), hexyl 2-methylbutanoate (RTI = 1418), 2-phenethyl acetate (RTI = 1788), ethyl phenylacetate (RTI = 1823), and ethyl cinnamate (RTI = 2102). The RTI for standard polar columns was obtained from PubChem. All chemicals were purchased from Sigma-Aldrich.

A pooled white wine (mixture of Riesling, Müller-Thurgau, Manzoni Bianco, Sauvignon Blanc, Veltliner, and Gewürztraminer) was used to confirm the performance of this targeted analysis approach in real-life analysis. The pooled white wine was diluted 10 times to obtain the result of the unsaturated condition. To create the saturated condition, for each 0.5 mL of diluted white wine, 10 μL of the 200 mg/L standard solution was added.

### Sample Preparation and Injection

Standard solutions were analyzed by the liquid injection mode. 1 μL was injected for each sample. Liquid injections were performed with a Gerstel MPS2 autosampler monitored by ChromaTOF (LECO Corporation, St Joseph, MI, USA). Pooled white wine samples were analyzed by solid-phase microextraction. 0.5 mL of the sample was mixed with 0.5 g of NaCl in a 20 mL headspace vial. Before the analysis, 25 μL of the internal standard solution (2-octanol in ethanol at a concentration of 2 mg/L) was added. A 2 cm 50/30 μm DVB/CAR/PDMS fiber was used (Supelco, Bellefonte, PA, USA), conditioned according to the manual. Other details can be found in the literature.^17^ Each sample was analyzed in three replications.

### Instruments and Data Processing

Samples were injected into the GC×GC system (Agilent 7890 A, Agilent Technologies, Santa Clara, CA) in the splitless mode. Separation was performed by a VF-Wax column (100% polyethylene glycol; 30 m × 0.25 mm × 0.25 μm, Agilent J&W Scientific Inc., Folsom, CA) in the first dimension and Rxi-17Sil MS (1.50 m × 0.15 mm × 0.15 μm, Restek Bellefonte, USA) in the second dimension. A nonmoving quad jet dual-stage thermal modulator was used to couple the two columns. The MS signal was obtained using the Pegasus IV time-of-flight (TOF) mass spectrometer (LECO Corporation, St. Joseph, MI, USA).

The applied column flow was 1.2 mL/min. The oven temperature was programmed from 40 °C (4 min holding time) to 250 °C at a rate of 6 °C/min. The final temperature of 250 °C was reached and then maintained for 15 min. The temperature offsets of the second oven and modulator were set at +5 and +15 °C, respectively. Within the 7 s-modulation time, 1.4 s was used for a hot pulse. In the beverage study, such a long modulation time is necessary to clear the second column before the next modulation cycle. The transfer line was kept at 250 °C. The TOF mass spectrometer was operated in the electron ionization mode at 70 eV. The ion source temperature was 230 °C. The acquisition frequency was 200 Hz within the mass scan range from 40 *m*/*z* to 350 *m*/*z*. The detector voltage was −1341 V. A 7 min acquisition delay was applied for liquid injection.

GC×GC–MS data acquisition and untargeted processing were achieved using LECO ChromaTOF (Version 4.22). ChromaTOF performs the *m*/*z* alignment automatically before the signal processing, rounding the measured *m*/*z* value to the nearest integer. The baseline offset was 1 for the signal preprocessing. The expected peak width was 0.8 s. For the peak picking, the signal-to-noise ratio was 100. Also, a minimum of five ion fragments were required.^18^ Peak annotation was achieved by comparing the constructed mass spectrum to the reference spectrum in the database.The MS databases used were NIST/EPA/NIH 11, Wiley 8, and FFNSC 2. The mass spectrum similarity threshold for the peak annotation was 700. Intermeasurement alignment was performed by the Statistical Compare package (ChromaTOF build-in) to improve the peak annotation and quantification.

### Saturated Peak Subtraction

Unsaturated and saturated chromatograms were obtained from the analysis of diluted pooled white wine and diluted pooled white wine plus standard solution (10 μL and 200 mg/L), respectively. With the diluted wine, there is no saturation occurring in the chromatogram, and adding the concentrated standard solution, the region of saturation is well-controlled. The saturation-subtracted chromatogram is the output of the proposed target method applied to the saturated chromatogram.

## Results and Discussion

### Detector and Column Saturation

**Figure 1:**
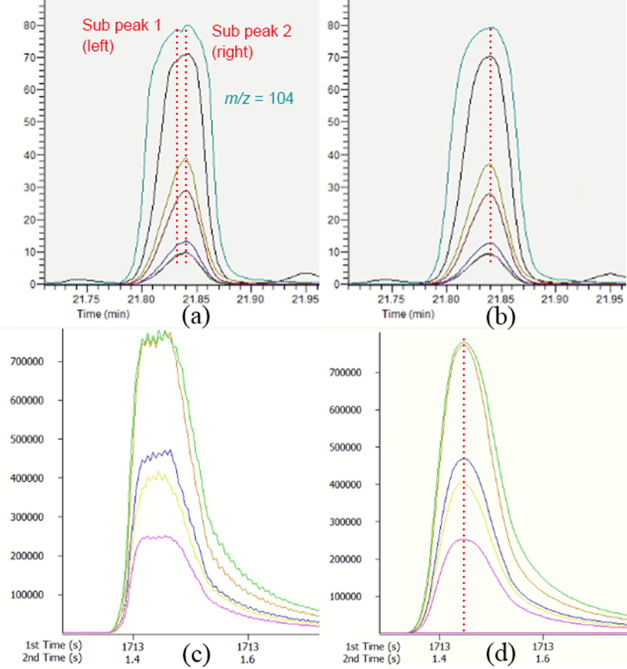
Chromatograms of 2-phenylethyl acetate by Xcalibur, Thermo Scientific: (a) detector saturation and (b) Gaussian smoothed signal of (a). The applied smoothing window was seven points. Chromatograms of methyl anthranilate by ChromaToF, LECO: (c) column saturation and (d) moving averaged signal of (c). The applied smoothing window was 19 points.

Overconcentration may lead to column and detector saturation and, consequently, to peak picking errors. An example of the possible issues is presented in Figure 1. The plot illustrates the effects of the detector and column saturation on the peak shape in the presence of highly concentrated 2-phenylethyl acetate and methyl anthranilate, respectively. In the case of detector saturation (Figure 1a), the ion channel at 104 (also noted as *m*/*z* = 104) shows a clear flat region close to the apex point. The presence of this flat area has two immediate consequences. First, the apex of this *m*/*z* is not in line with the ones of other unsaturated ion signals associated with the same neutral compound. Second, the presence of more than one local maxima on the ionic signal results in the “splitting” of the peak into two sub-peaks. Sub-peak 1 contains the signal at *m*/*z* = 104 only. Due to the absence of the higher intensity peak from the compound spectrum, mass spectrum similarity-based annotation on this peak will most likely be incorrect. Sub-peak 2, instead, contains the signal from all aligned ion channels, including *m*/*z* = 104. The annotation here will be correct, but since a part of the signal from *m*/*z* = 104 is assigned to sub-peak 1, if this ion is to be used for the quantification, the peak area will be underestimated.

The effects of column saturation are presented in Figure 1c. In this case, the signal intensity is below the detector saturation limit (10^6^), but the signals of all the ion channels are flattened at the top with noise from detector oscillation. Under these circumstances, the apex alignment result is based on random electronic noise, and the annotation and quantification errors are unpredictable.

The conventional solution implemented in commercial software to cope with saturation effects is to apply signal smoothing. Figure 1b shows the smoothed signal of the saturated ionic channels (Figure 1a). After the smoothing, the situation is apparently improved as the apexes of all channels are now aligned. Nonetheless, generalized smoothing of the ionic signals also presents severe drawbacks. The size of the smoothing window must be well-adjusted to counterbalance the effects of saturation, though, at the same time, as small as possible. In our example, the required minimal smoothing window size is seven data points, which is nearly one-fifth of the peak width. In the case of column saturation, because of the larger flattened region, a wider smoothing window is required. As an example, to align the signals from all the channels in Figure 1c, the size of a moving average window has to be larger than 19 points (Figure 1d), which is half of the peak width. Such a severe smoothing will result in the loss of small peaks, which can be detrimental for the coverage of the overall analysis. To counterbalance this effect, it is necessary to treat the signals of major and minor compounds differently.

### Targeted Data Processing

**Figure 2:**
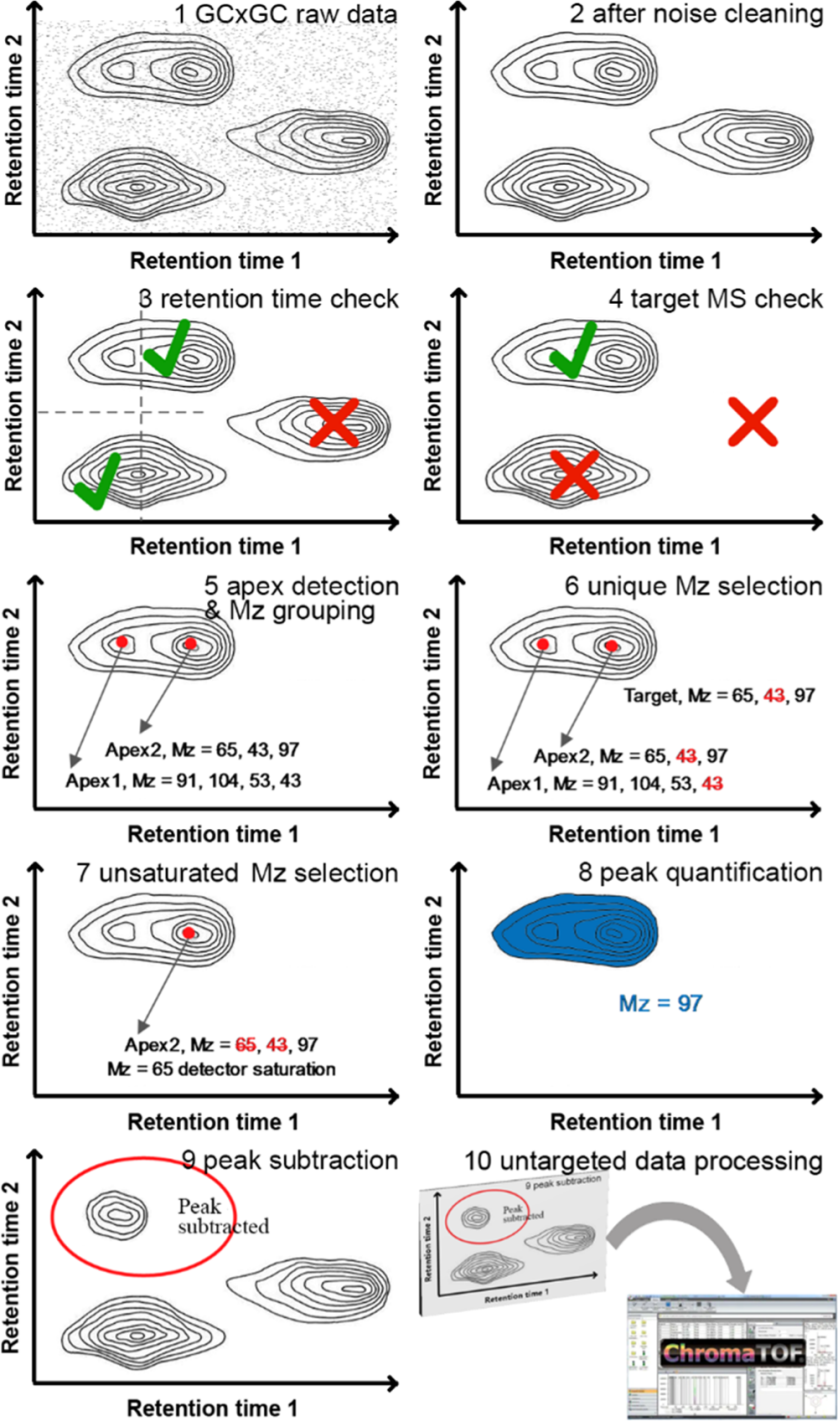
Target guided data processing flowchart.

In a metabolomics study, the nature of the samples is clearly defined, and the major compounds that have the potential to lead to saturation are almost invariably known. Their RTIs and MS information can then be collected and stored in a matrix-specific library. This library will have a crucial role in the proposed two-stage approach. Figure 2 illustrates the scheme of the proposed workflow. The workflow starts from the raw GC×GC data in the netCDF format. These “raw” data are exported from ChromaTOF after automatic *m*/*z* alignment. The proposed workflow starts after the removal of background noise when the regions corresponding to “true” signals are detected in the 2D chromatographic plane (step 2). These regions of “interests” (ROIs) are then matched with the reference library to annotate the targeted compounds (steps 3 and 4). Due to the presence of potential coelution within the annotated signal region, it is necessary to detect how many peaks are actually present within each ROI (step 5). For each targeted compound, an unsaturated *m*/*z* that is exclusively present in the reference library is selected for peak quantification (steps 6–8). The signal of the annotated peak is then subtracted from the chromatogram (step 9). The rest of the chromatogram still contains the signal of unannotated peaks. It will be analyzed by a downstream untargeted data processing approach (step 10). The steps 2–9 are performed by a set of bioinformatic scripts developed in R. The steps are presented in more detail below.

To construct the retention time and MS library for targeted compounds, pure standards have to be analyzed at the level just below saturation to obtain as much signal as possible. After the data processing, the normalized mass spectrum of identified peaks can be added to the ChromaTOF library. Retention time is manually added to the compound’s label. The ChromaTOF library can then be converted to a text file (SDF format) by lib2nist (NIST MS Search). This text file is ready to be processed by R. Hereafter, the workflow steps are presented in detail.

(1) *Data export*. GC×GC–MS data have to be exported from GC×GC instrument control software (step 1). In this study, GC×GC data were acquired by ChromaTOF and then centered to the integer mass-to-charge ratios to compensate for potential mass shifts. ChromaTOF was also used to save the preprocessed data in the netCDF format.
(2) *ROI detection*. Noise estimation and signal region detection were performed on the total ionic chromatogram (TIC). The background was removed by implementing an approach explicitly developed for GC×GC data.^19^ The whole chromatogram is split into a number of sub-chromatograms based on the first-dimensional retention time. For each segment, the algorithm identifies a set of local minima. The standard deviation (SD) of the signal in the vicinity of the minima is used to estimate the noise SD, which is then used to give a robust estimate of the local noise level. This method is rather robust since it does not assume any statistical property of the entire background. In our experiment, if the signal is higher than 1.7 times the local maximum electronic noise, it is considered as the true signal. The regions containing true signals were labeled as ROIs. It is important to point out that due to coelution, one ROI may contain more than one peak.
(3) *Library matching*. ROIs were matched with the library on the bases of their positions in the GC×GC plane (step 3). A rectangular tolerance window was selected in the GC×GC plane (30 s in the first dimension and 2 s in the second dimension of the chromatogram). The ROI was “flagged” as a potential peak region if it was partially overlapping the tolerance window around a library peak. Each potential peak region corresponding to a targeted compound was then annotated according to the mass spectra stored in the library (step 4). Annotation criteria will be explained in the Annotation Criteria section.
(4) *Coelution check and m/z grouping*. At the end of the previous step, a region is flagged as “annotated” if it contains at least one peak of the library compounds. To check the purity of the annotated peak regions, a 2D apex detection algorithm was applied.^11^ For each *m*/*z*, a 2D sliding window will traverse each pixel of the annotated region, looking for local maxima. The size of the sliding window should be large enough to ignore the effect of detector oscillation. We used three data points (cover two modulation cycles) in the first dimension and 21 data points (cover 20 acquisition period) in the second dimension. In our experiment, a location showing more than 15 aligned ion apexes was considered a true peak. This threshold was fixed, striking a balance between the number of ionic traces that could show matching apexes by chance (usually two/three) and the number of *m*/*z* collected in the MS library of targeted compounds. At this point, if an annotated peak region contains only one true peak, it is flagged as a pure peak region. Otherwise, it is a region of mixed peaks. It is important to point out that when severe column saturation occurs, signal smoothing could be applied to the second chromatographic dimension. Whether to use a smoothing process or not should be assessed by preanalyzing the standards at the expected concentration. Our study applied moving average smoothing with 101 data points and 201 data points windows on the second dimension for the 200 and 2000 mg/L standard solutions, respectively. For a pure peak region, the unique *m*/*z* selection (step 6) is skipped. Our interpretation continues with the region of mixed peaks.
(5) *Identify unsaturated unique m/z*. For a region consisting of more than one peak, it is necessary to identify unique ion channels of the annotated compound (step 6) in order to proceed to quantification and signal subtraction. In the previous step, the apexes were detected at each *m*/*z*. After the apex alignment, for each “true” peak, the aligned ion channels are known. Based on the mass spectra of the target compound recorded in the library, *m*/*z* belonging to the target compound are checked one by one. If an *m*/*z* appears on only one peak, it can be judged as the unique *m*/*z* for the target compound. If a coeluting compound shares the same ion channels with the targeted compounds, it is not possible to obtain a unique *m*/*z*. In this case, the signal in this peak region will be untouched by the targeted approach and sent downstream to the untargeted workflow. It is worth noting that if two coeluting compounds are not distinguished on the mass spectrum and the peak shape is disrupted by the saturation of the detector or column, distinguishing these peaks is not possible. The intensities of the unique ion channels are collected at the peak apex and are flagged for saturation. An *m*/*z* is considered saturated if its intensity is higher than the manually defined detector saturation level (step 7).
(6) *Peak quantification and subtraction*. The most intense unsaturated unique *m*/*z* is then used for peak quantification (step 8). Peak quantification is achieved by integrating the ionic signal of the selected *m*/*z* over the entire annotated ROI.^20^ After peak quantification, the MS signal of the targeted compound is subtracted from the chromatogram (step 9). Since the relative intensity of each *m*/*z* associated with the targeted compound is available in the library, the intensity profile of the unique *m*/*z* can be used to calculate the overall profile associated with the targeted compound. This overall profile is finally subtracted from the ROI. The leftover signal comes from the untargeted compound(s). For saturated ion channels, the estimated intensities are greater than the experimental intensities recorded in the chromatogram. The subtracted residual is below zero and is replaced by zero.
(7) *Untargeted processing*. The signal remaining in the chromatogram will be reloaded to ChromaTOF for untargeted processing (step 10).

### Annotation Criteria

Targeted compounds are identified in the ROIs by matching the experimental signals with the standard library. In our approach, four criteria must be satisfied for a successful peak annotation: (1) the difference in RTIs should not exceed an experiment-specific threshold, (2) the similarity between the spectra should be high, (3) the ion channels with the highest intensity in the database should always be present in the sample, and (4) the rank of the ion channels with the highest intensity should be preserved in the sample.

The RTI and mass spectrum similarity are widely used in untargeted approaches and well-known to every chromatographer. In routine analysis, the acceptable tolerance in the retention time dimension for each experimental setting is known and can be assessed by checking the retention times obtained from past analyses. The RTI, instead, can be extracted from MS databases. It is important to notice that the deviation of the indices value present in the public database is in general much larger than the deviation of the experimental retention time measured on a specific instrument. With the RTI, more peak regions may be selected for further mass spectrum annotation. This will increase the uncertainty on the final result. In this study, we proposed several general criteria for saturated peaks. However, the annotation criteria can be compound-specific. More criteria are available in a previous publication.^21^

In our algorithm, mass spectrum similarity is calculated by a composite optimized dot product algorithm.^22^ This algorithm uses a small computation power and does not require programmatic access to the large MS database. The calculated mass spectrum similarity value ranges between 0 and 1000. Since a large peak is less likely to be covered by small peaks, we believe that the peak of the targeted compound is at least partially separated from that of the coelutes. The minimal mass spectrum similarity threshold used in our study was 995. This threshold can, however, be manually tuned by the user. As usual, a more liberal threshold will improve the annotation efficacy at the price of potential false results.

In our approach, we decided to add two additional rules which take into account the relative intensity, the presence, and the order of the most intense ion channels to these two well-established criteria. These rules increase the confidence in annotation when saturation and coelution occur. To be comparable with common ChromaTOF procedures, the examined *m*/*z* number was 5 in our study.^18^

The first criterion relies on the relative intensity of the most intense ion channels in the MS library in the sample. Since the targeted peak is the major peak, its intense ions must be the most intense in the mass spectra by at least some pixels in the GC×GC peak region. This criterion is robust to the interference caused by detector and column saturations. The second criterion requires not only that the intense ion channels are present but also that they are showing the same rank that they have in the library spectrum. The rank may be altered by the signal of coeluted compounds. However, our targeted approach focuses on the major metabolites in the sample. It is unlikely that the mass spectrum of a major compound will be largely altered.

In addition, in terms of similarity, presence, and sequence of the mass spectra, the comparison is based on the single mass spectrum. As long as one data point (or one pixel) in the peak region passes the check, positive feedback is returned for the whole region. One of the advantages of this annotation is the use of all mass spectra of a single peak region. It avoids the apex detection, ion channel grouping, deconvolution, and mass spectrum construction for the apex point. Therefore, the annotation results are not affected by the errors in these processes.

### Improve the Annotation and Quantification for Targeted Peaks

**Figure 3:**
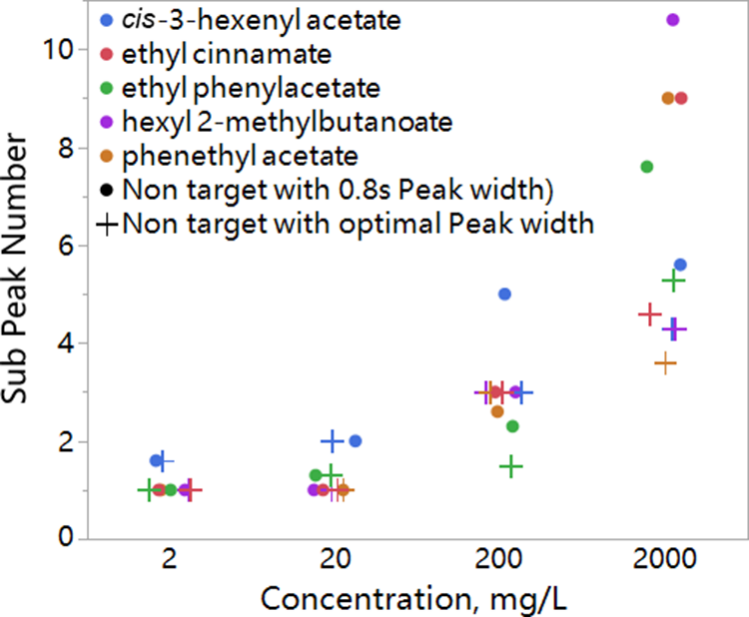
Compare the sub-peak number for untargeted approaches with the fixed and optimal peak width.

The first benchmark of the proposed pipeline was performed on a dilution series of standard compounds (2, 20, 200, and 2000 mg/L). The results of the analysis were compared with the ones obtained by a full untargeted approach. It is well-known that choosing a suitable peak width is important for an untargeted approach, so the untargeted pipeline was run with both a standard 0.8 s and an optimal peak width. The optimal peak width can be estimated by looking at the chromatogram manually. As expected, when the column was saturated with 200 and 2000 mg/L standard solutions, peaks were split into a few sub-peaks by deconvolution (Figure 3). The number of sub-peaks detected by the different processing pipelines for the different compounds injected at different concentrations is shown in Figure 3. Severe overdeconvolution may lead to incorrect quantification. In our study, with the 2000 mg/L standard, the peak of phenethyl acetate is spilt into several sub-peaks, and *m*/*z* = 48 was chosen for the quantification. This *m*/*z* does not exist in the library-recorded mass spectrum of phenethyl acetate.

**Figure 4:**
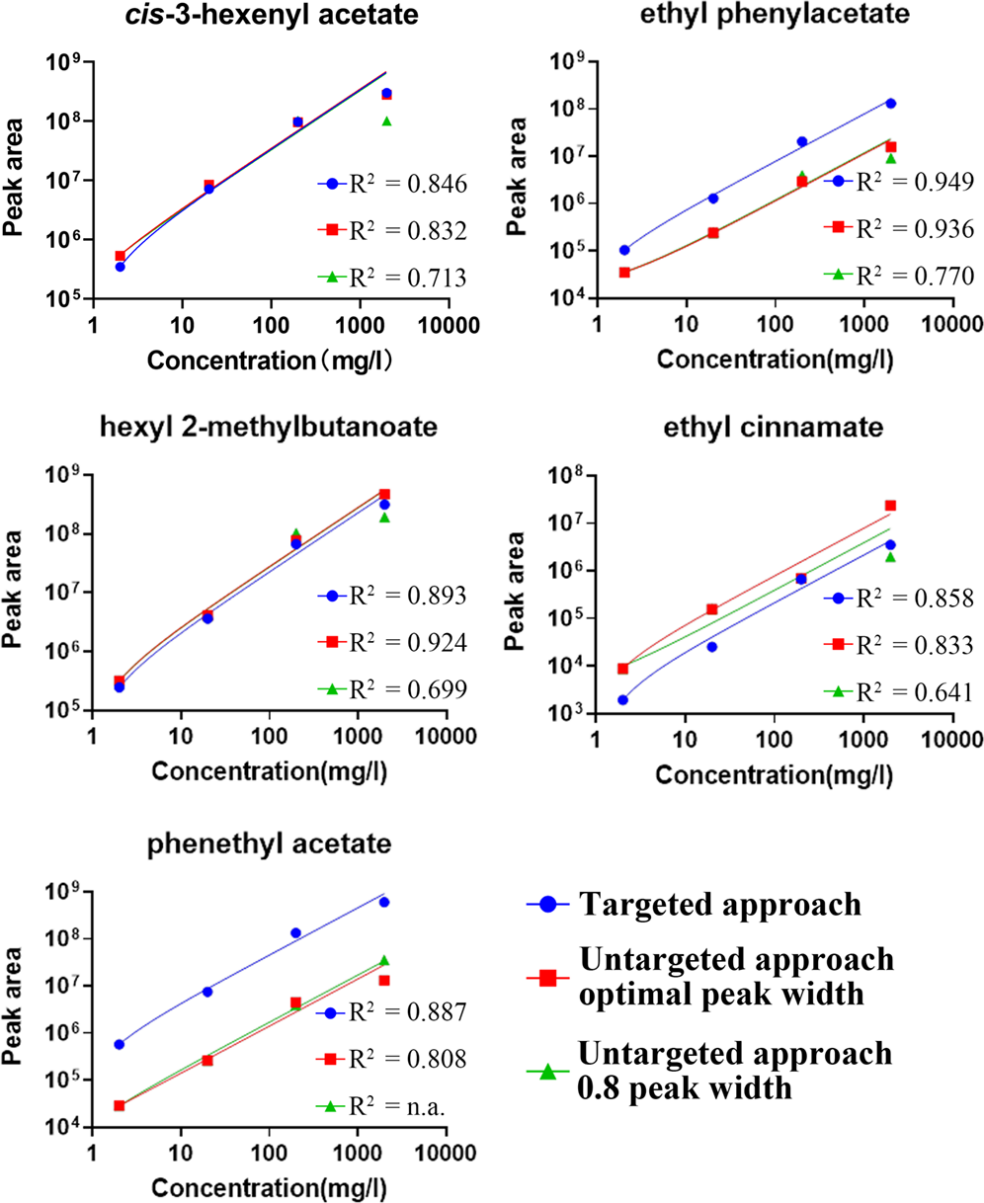
Comparison of the weighted linear regression for standards quantified with the proposed targeted approach and untargeted approaches.

When a suitable peak width is applied, overdeconvolution is significantly reduced, as shown by the low hanging cluster of crosses in Figure 3. The peak list reported by ChromaTOF usually consists of one major peak and several sub-peaks for each compound. All peaks are quantified using ion channels, which are easily found in the MS library. A weighted linear regression process (by GraphPad Prism) was performed to examine the linear relationship between the peak area and concentration of the detected major peaks, and the results are shown in Figure 4. With the proposed targeted approach, linearity (measured in terms of *R*^2^) is generally improved, with the only exception of hexyl 2-methylbutanoate, where both the targeted approach and peak width optimized untargeted approach provide high linearity. This means that the samples can be quantified more accurately in the concentration range tested. It is worth noting that in this case, we are using pure standard solutions. It is not difficult to pick up the correct peaks manually. However, for real samples, manual peak picking is still tricky and subjective.

In addition, with the proposed targeted approach, peak splitting does not exist. Because the entire signal region is used to calculate the peak area, differences in quantification results only arise from background noise cleaning. This also improves the analytical reproducibility and the relative SD across multiple injections (Table 1). Besides, the proposed targeted approach does not require any manual supervision. It reduces the labor cost and human error during the analysis of a complex sample.

**Table 1:** Compare Peak Area Relative SD % Among Untargeted Data Processing with a 0.8 s Peak Width (p.w.) and a 3 s p.w. and Target Data Processing for the 2000 mg/L Standard Solution

compounds	untargeted 0.8 s p.w.	untargeted optimal p.w.	targeted
*cis*-3-hexenyl acetate hexyl	3.0	23.2	0.9
2-methylbutanoate	54.3	1.7	1.5
phenethyl acetate	n.a.^a^	3.9	2.3
ethyl phenylacetate	38.5	22.7	3.9
ethyl cinnamate	26.2	2.3	1.5

a When column saturation occurs. For phenethyl acetate, peak quantification is not possible with an untargeted approach and a 0.8 s peak width applied.

### Improve the Peak Annotation for Untargeted Coeluting Peaks

**Figure 5:**
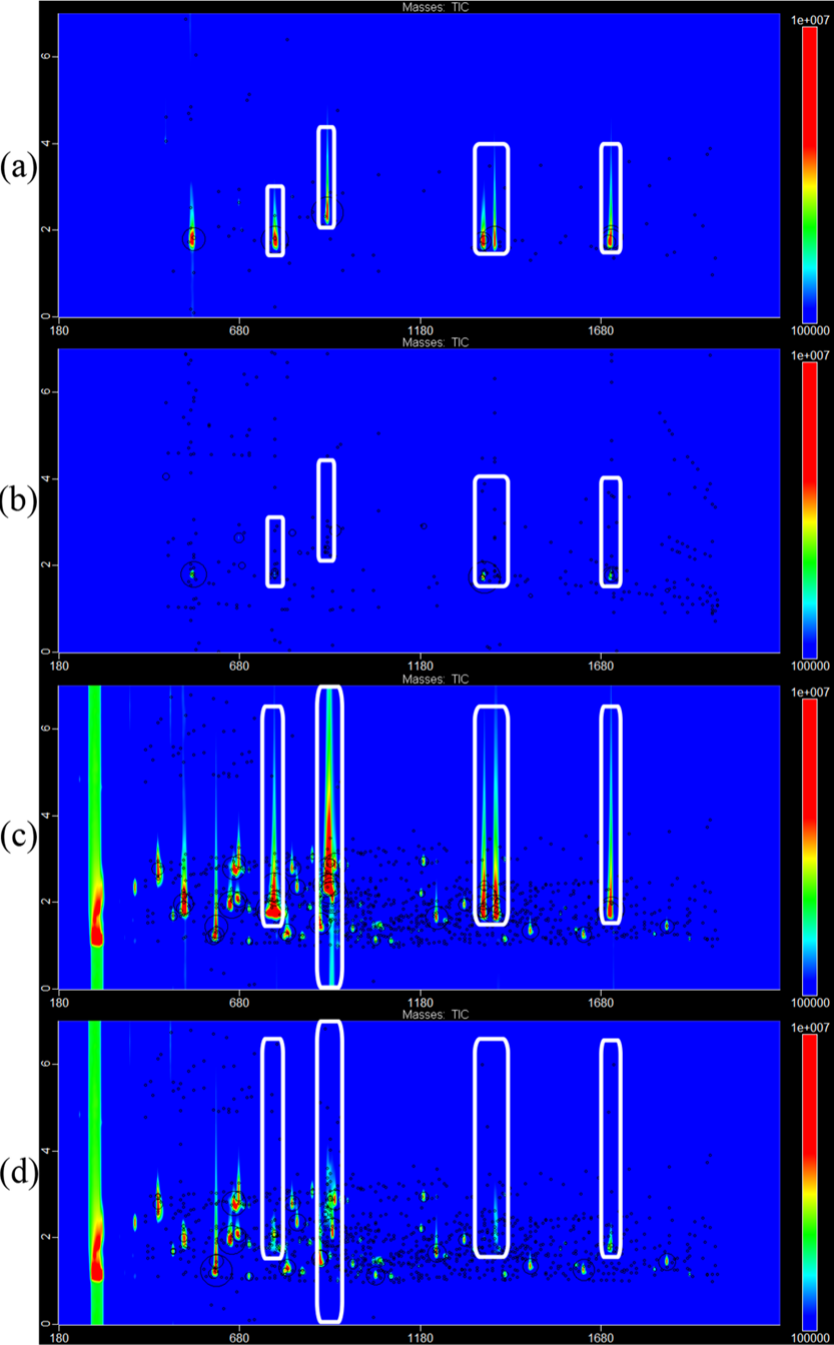
Peak signal (marked by white label) subtraction results. (a) Chromatogram of the 200 mg/L standard solution; (b) peak signal subtracted chromatogram of (a); (c) chromatogram of 10 times diluted mixed white wine plus 10 μL of the 200 mg/L standard solution; and (d) peak signal subtracted chromatogram of (c), obtained by ChromaToF, LECO.

Targeted peak signals were subtracted from the original chromatogram according to the peak profile estimated with the experimental profile of unique *m*/*z*. The subtraction result is displayed in Figure 5 for the solution of standards (top panels) and for the wine sample. It can be seen clearly that peak signals are mostly removed in both standards and pooled wine chromatograms. There are, however, some small artifacts which arise from the two phenomena. First, the intensity of the mass spectrum of the targeted compound in the library is not recorded with a sufficient accuracy by ChromaTOF. Relative intensities for each *m*/*z* are, indeed, recorded as integers. This lack of accuracy propagates through the estimation of the signal that has to be subtracted from the raw data, leading to a mismatch between the estimated profile and the true profile of each peak. The other source of incomplete signal subtraction is the procedure applied during peak region detection. For convenience, the peak region was detected based on the TIC. By summing up the signal of each *m*/*z*, the electronic error is also enhanced. Detecting the peak region on every *m*/*z* and then merging regions will provide more accurate peak region detection.

Predictably, artifacts resulting from incomplete signal subtraction may affect the untargeted peak picking of coeluted compounds. To assess this effect, chromatograms of unsaturated (diluted wine), saturated (diluted wine plus standards), and subtracted saturated peaks were analyzed by the untargeted approach of ChromaTOF. It is important to remember that the saturated chromatograms were recorded on a set of samples created by adding diluted wine with concentrated standards, so saturation occurs only in the area where the standards appear. The saturated areas are marked in white squares in Figure 5. The four areas account for the five saturated compounds because the second right white square contains two saturated peaks. A comparison of the results of the annotation in the presence of saturation and the results of the proposed pipeline is presented in Table 2.

**Table 2:** Comparison of Untargeted Data Processing Results among Unsaturated, Saturated, and Saturation-Subtracted Chromatograms

	annotated peak	NIST dot product similarity, average
unsaturated	28	839
coeluting with saturated standards	17	806
saturation subtracted	20	841

Depending on the location and size of the saturated region, 28 peaks which were coeluting with the five saturated standards were annotated in the diluted wines. There are in total 57 peaks detected. However, interclass peak alignment with heavy saturation is difficult. Many false detected peaks are present when saturation occurs. It is only possible to trace the annotated peaks. Table 2 also shows their average matching score with the NIST library: 839. Out of these 28, 17 were also identified in the samples spiked with the standards. The presence of saturated peaks is then reducing the annotation capacity of ChromaTOF of around 10%, with an overall degradation of the matching score (which decreases to 806). When the annotation pipeline is applied on the samples where the saturated peaks were removed, the number of matchings increased to 20, with the overall matching score increasing back to 840. Remarkably, only one of the annotated peaks has the similarity score slightly below 700. The fully annotated peak table, MS examples, and information of the original peak table are available in the Supporting Information.

Even if after the subtraction of the saturated peaks, the overall annotation of the untargeted pipeline improved, the subtraction of the signals of the target peaks from the saturated chromatograms did not completely eliminate the interference of the coeluting compounds. Of the 28 annotated peaks in the diluted wine, only 20 have been found. This may be attributed to the artifacts introduced by the subtraction (see the previous discussion about the standard analysis). However, the results suggest that our proposed saturation subtraction approach has the potential to reduce the interference of large saturated peaks with the coeluting peaks during peak picking. The mass spectra constructed after the signal subtraction showed an improvement by 30 units in the average similarity. The improvement in this similarity is better than expected for the actual peak annotation because the improvement occurs mainly for the peaks with low similarity. It is important to increase the similarity of “bad” peaks above 700, which is the mass spectrum similarity threshold used for annotation in most recommended untargeted approaches.^18^

## Conclusions

Metabolite concentrations differ from one another by orders of magnitude. This poses difficulties during metabolite profiling. Errors can occur during peak picking and mass spectrum construction when the detector and/or column are saturated with major metabolites. In metabolomics studies, the nature of the sample is well-defined, and the major compounds that have the potential to cause saturation are almost invariably known. Data processing results can be improved by a two-stage data processing strategy that will incorporate a targeted data processing and cleaning approach upstream of the “standard” untargeted analysis. Our experiments show a significant improvement in the annotation and quantification results for targeted saturated compounds. After subtracting the signal of saturated compounds, the mass spectrum construction was improved for coeluted compounds. Incomplete signal subtraction may occur. It leads to the detection of false positive peaks or to interferences with the construction of mass spectra of codiluted peaks. High-resolution MS libraries and more accurate peak area detection methods should be tested for further improvement.

## Supporting Information Available

R scripts and Instructions. Files used in the exemplar methods and supporting documentation available via a repository at https://github.com/PenghanZ/target-guided-data-processing-approach-to-GCxGC-analysis (PDF)
