# Supplementary material for: Application of a Target-Guided Data Processing Approach in Saturated Peak Correction of GC×GC Analysis
Source: Anal Chem. 2022 Jan 20;94(4):1941–8. doi: 10.1021/acs.analchem.1c02719 (PMC8811747; doi:10.1021/acs.analchem.1c02719)
Supplement: Supplementary file 1 — ac1c02719_si_001.pdf [file ac1c02719_si_001.pdf]

Supporting information **application of a target-guided data processing approach in saturated peaks correction of GCxGC analysis**

Penghan Zhang[a,b,*], Silvia Carlin[a], Pietro Franceschi[a], Fulvio Mattivi[a,b], and Urska Vrhovsek[a,*]

[a]Research and Innovation Center, Edmund Mach Foundation, Via E.Mach 1, S.Michele all'Adige, 38010, Italy

[b]Department of Cellular Computational and Integrative Biology (CIBIO), University of Trento, Via Sommarive 9, Povo, Trento, 38123, Italy

*Email: urska.vrhovsek@fmach.it, penghan.zhang@fmach.it

Table S 1 Untargeted data processing results of potential co-eluted peaks with the saturated standards from unsaturated, saturated, and saturation subtracted chromatograms

				Similarity		
Peak #	Name	CAS	R.T. (s)	Diluted wine	Diluted wine + standard	Standard signal subtracted
209	3-hexen-1-ol, acetate, (Z)-	3681-71-8	768 , 1.725	Standards		
214	1-pentanol, 3-methyl-	589-35-5	775 , 1.265	922	925	931
215	5-hexen-2-one, 3-ethylidene-1-methoxy-	55956-40-6	775 , 2.140	707		
216	linalool ethyl ether	72845-33-1	775 , 2.295	874	610	818
221	1,3,5-cycloheptatriene, 3,7,7-trimethyl-	3479-89-8	782 , 1.965	745		
222	3-heptanol, 3,6-dimethyl-	1573-28-0	789 , 1.500	806		
223	propanoic acid, 3-ethoxy-, ethyl ester	763-69-9	789 , 1.685	728		
225	heptanoic acid, ethyl ester	106-30-9	789 , 2.035	916	799	822
229	6-methyl-5-hepten-2-one	110-93-0	796 , 1.665	859	665	744
230	propanoic acid, 2-hydroxy-, ethyl ester	97-64-3	796 , 2.320	869	944	924
303	hexyl 2-methylbutanoate	10032-15-2	915 , 2.325	Standards		
304	tetradec-1-ene	1120-36-1	915 , 2.990	866	935	934
308	benzene, 1-methyl-4-(1-methylethenyl)-	1195-32-0	922 , 1.810	867	582	759
309	benzene, 1-methyl-4-(1-methylethyl)-	99-87-6	922 , 1.930	779	738	809
313	octanoate <ethyl->	106-32-1	929 , 2.150	930	902	874
316	linalool oxide <*cis*->	5989-33-3	936 , 1.625	924	819	870
317	benzene, 1-ethenyl-3-ethyl-	7525-62-4	936 , 1.810	897	892	860
318	ethanone, 1-(3-methylphenyl)-	585-74-0	936 , 1.940	825		
319	dodec-1-ene	112-41-4	936 , 2.885	919		
323	1-octen-3-ol	53907-72-5	943 , 1.345	716		886
326	terpineol <alpha->	98-55-5	943 , 2.425	751	714	748
328	acetic acid	64-19-7	950 , 1.010	894	894	894
329	1-heptanol	111-70-6	950 , 1.340	868	860	944
330	benzene, 1-ethenyl-4-ethyl-	567776	950 , 1.815	864	861	875
605	benzeneacetic acid, ethyl ester	101-97-3	1349 , 1.710	Standards		
609	succinic acid, butyl ethyl ester	67233-92-5	1356 , 1.755	853		820
611	ethyl 4-hydroxybutanoate	999-10-0	1363 , 1.275	805		797
612	2,6-octadien-1-ol, 3,7-dimethyl-, (E)-	106-24-1	1363 , 1.470	842		
614	bicyclo[3.1.1]hept-2-ene-2-ethanol, 6,6-dimethyl-, (1R)-	35836-73-8	1363 , 2.200	711		
626	acetic acid, 2-phenylethyl ester	103-45-7	1384 , 1.675	Standards		
628	tridecanal	10486-19-8	1384 , 2.125	905	905	825
629	damascenone <(E)-, beta->	23726-93-4	1391 , 1.965	851	662	689
743	2-propenoic acid, 3-phenyl-, ethyl ester	103-36-6	1699 , 1.710	Standards		

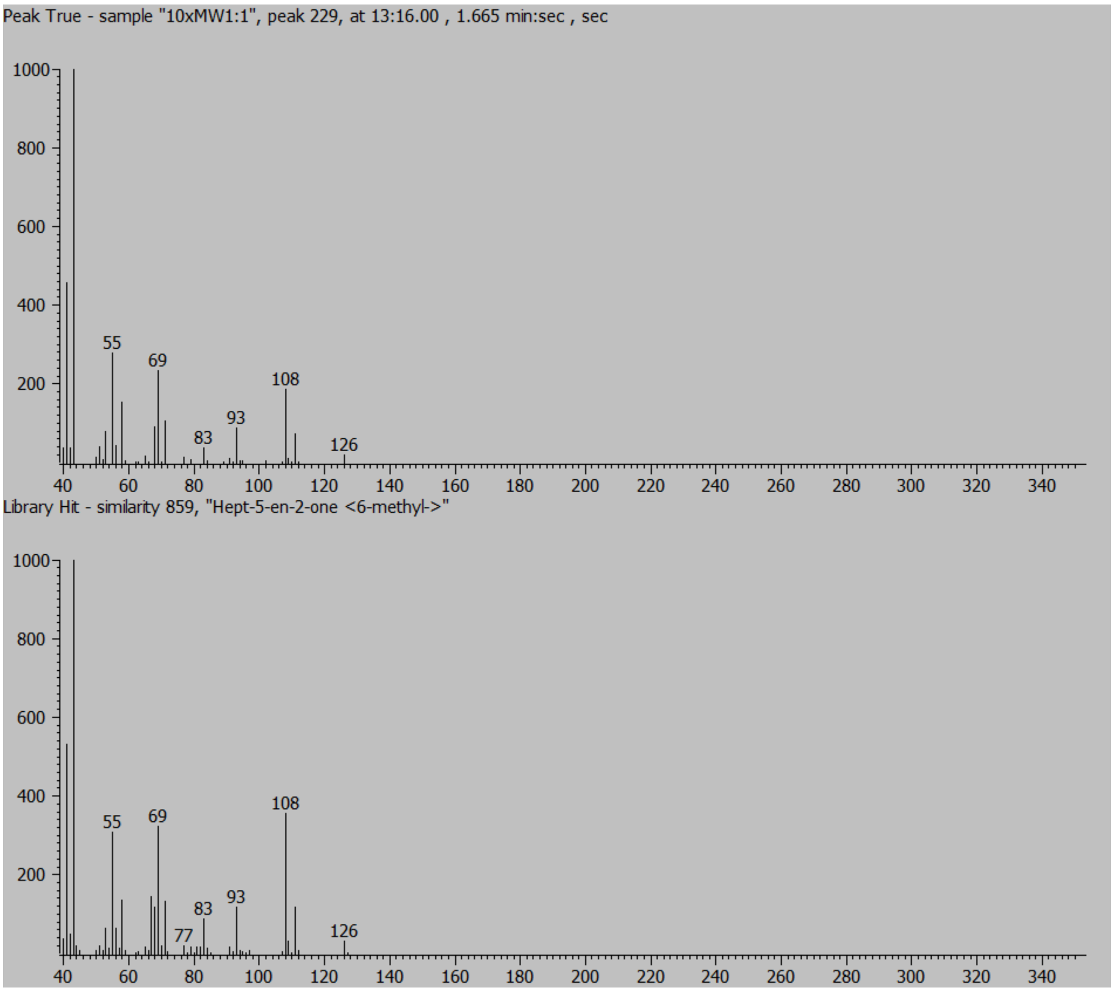

Figure S 1 the mass spectrum of 6-methyl-5-hepten-2-one under unsaturated condition

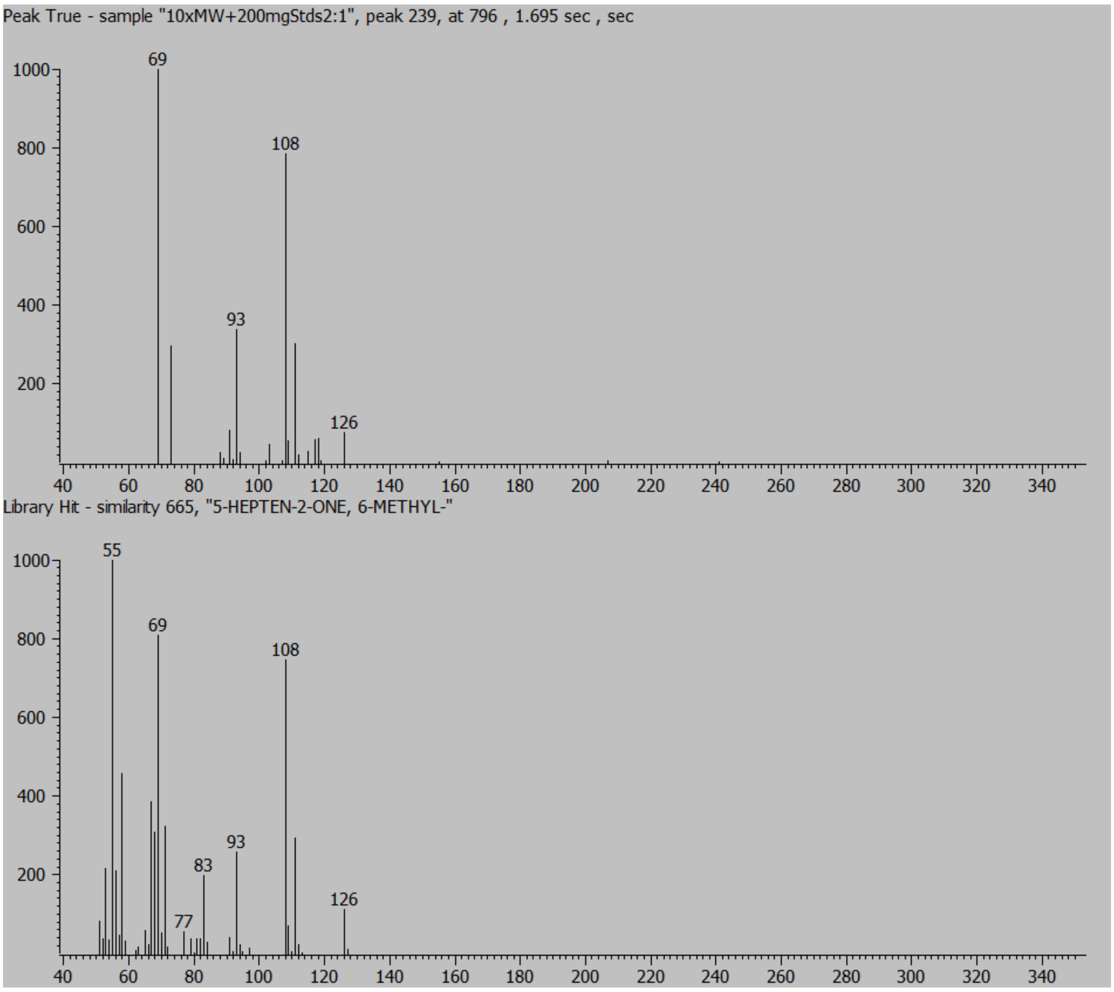

Figure S 2 the mass spectrum of 6-methyl-5-hepten-2-one affected by saturation

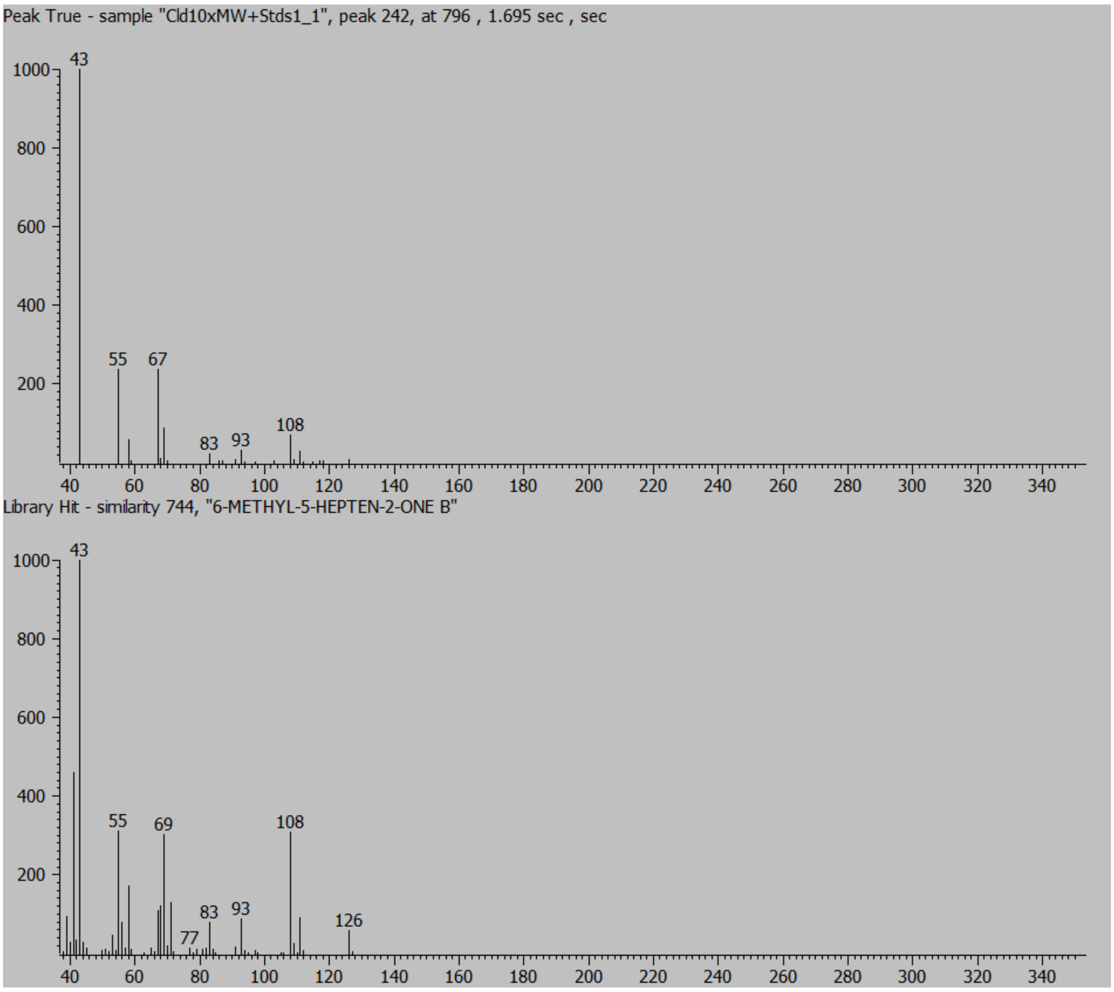

Figure S 3 the mass spectrum of 6-methyl-5-hepten-2-one after saturation correction

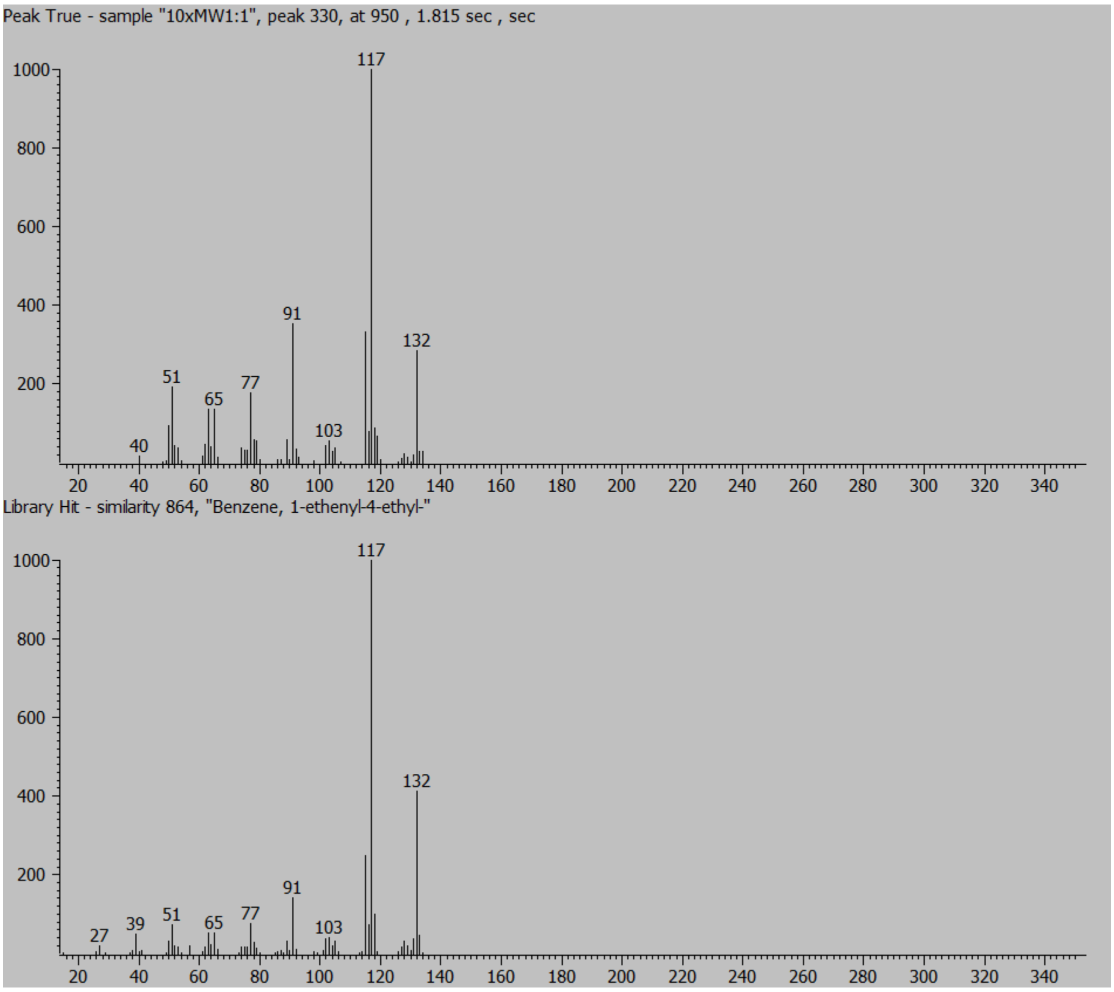

Figure S 4 the mass spectrum of benzene, 1-ethenyl-4-ethyl- under unsaturated condition

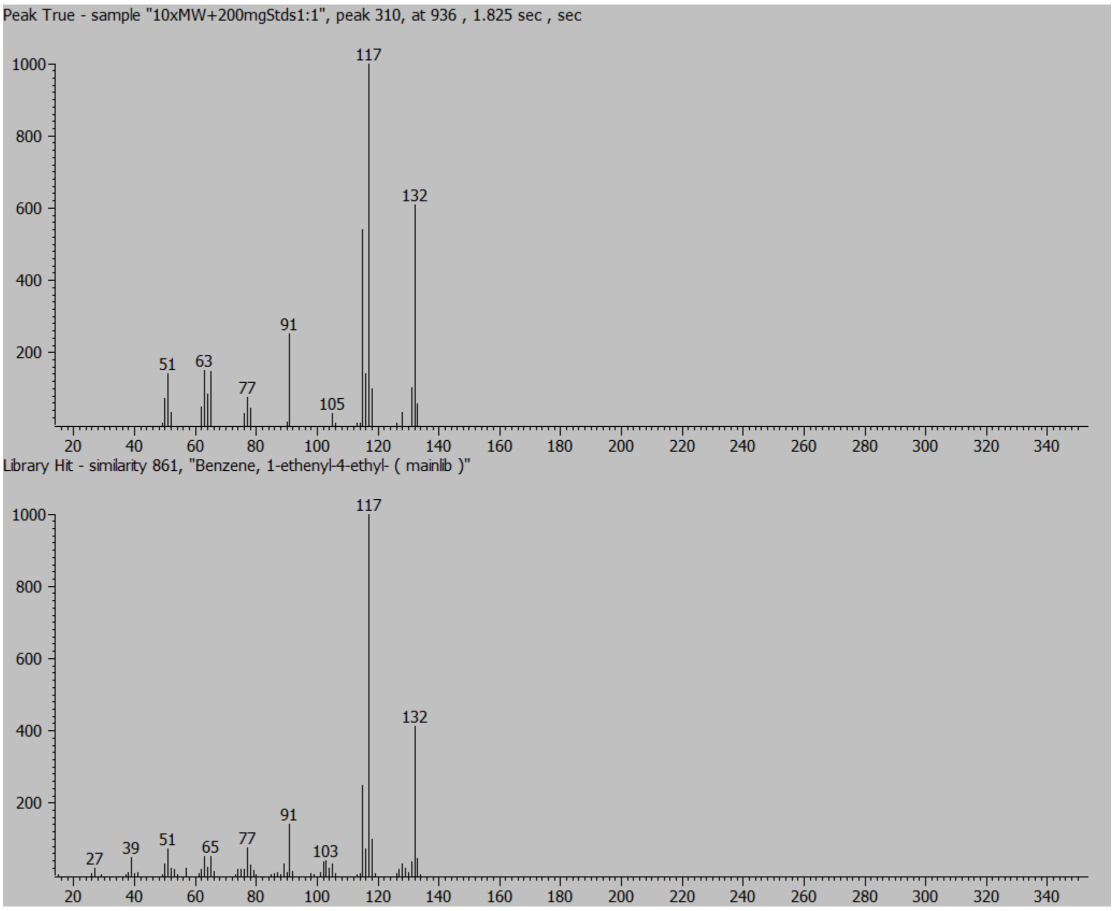

Figure S 5 the mass spectrum of benzene, 1-ethenyl-4-ethyl- affected by saturation

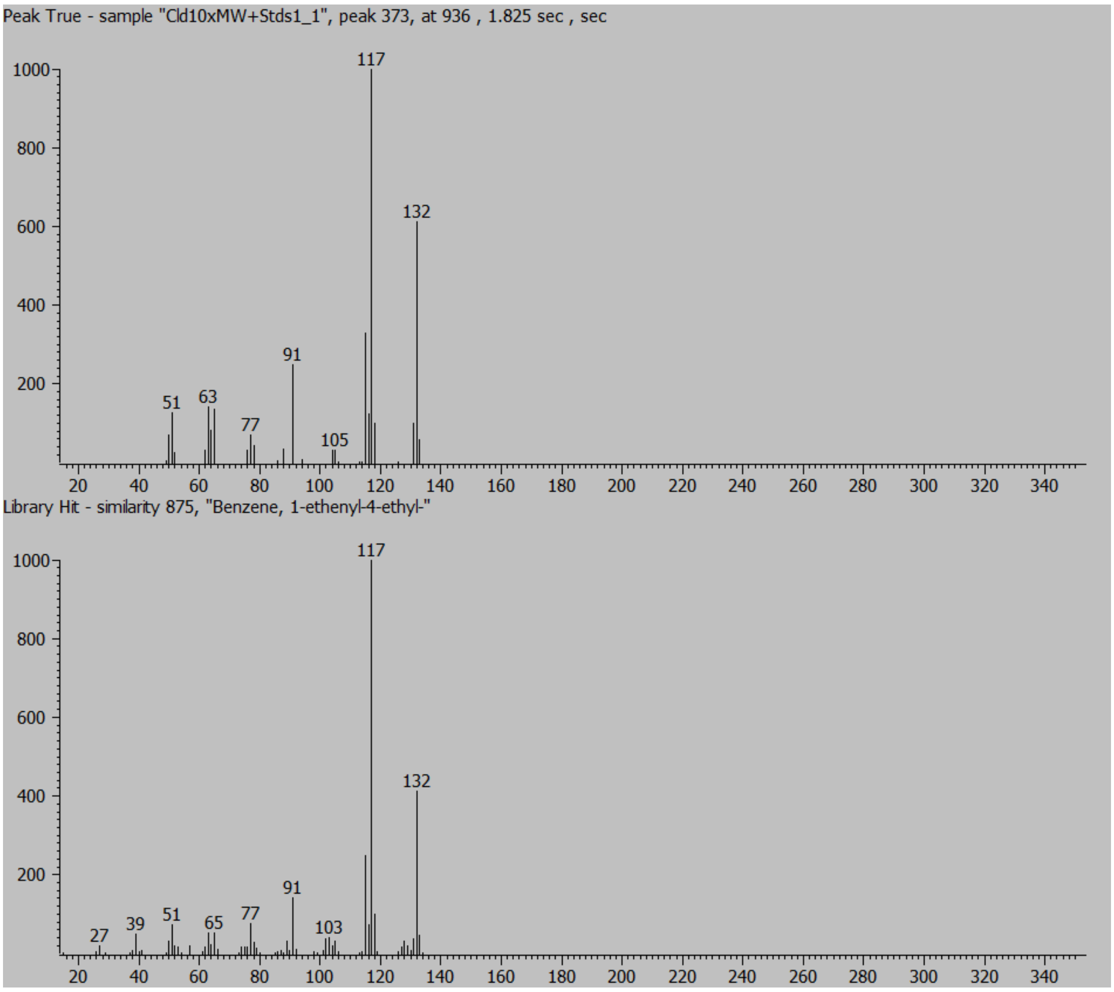

Figure S 6 the mass spectrum of benzene, 1-ethenyl-4-ethyl- after saturation correction

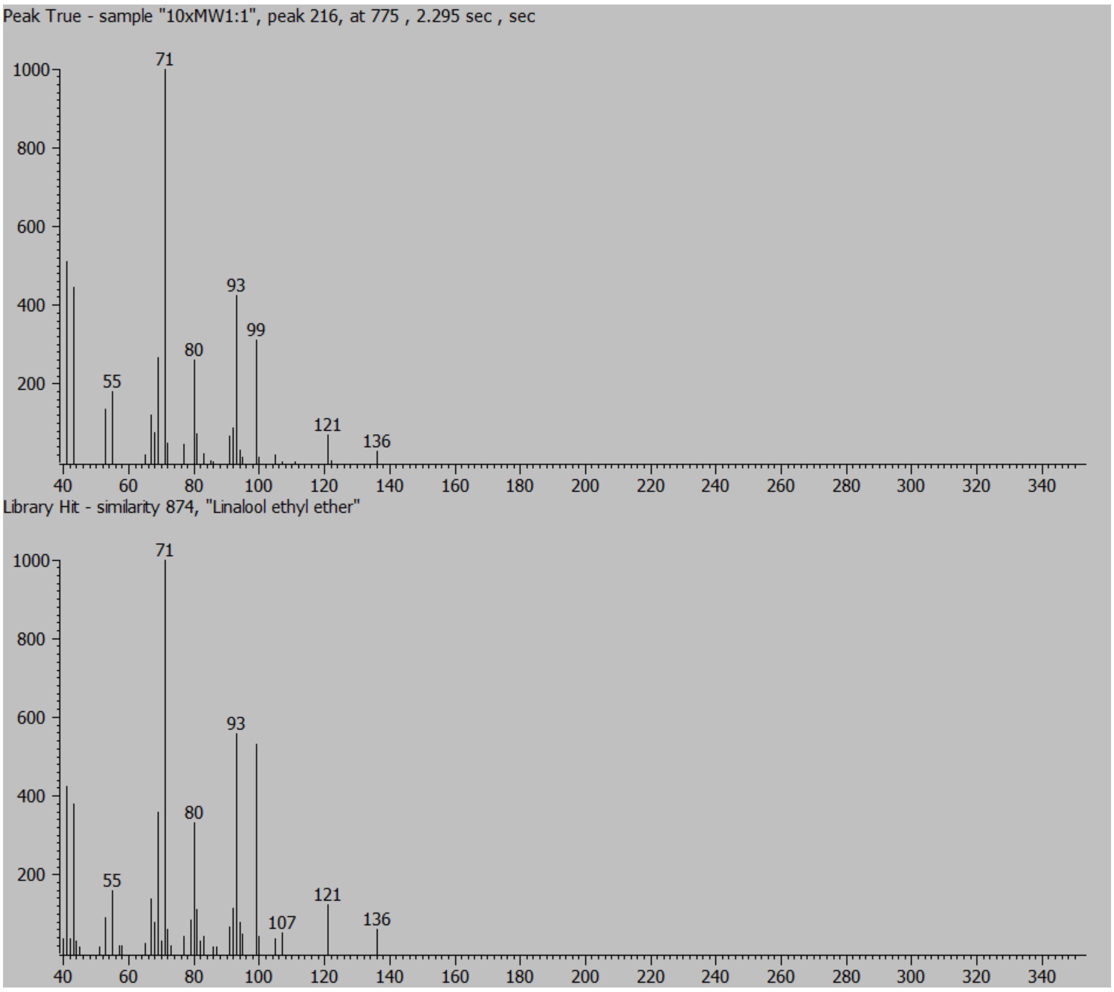

Figure S 7 the mass spectrum of linalool ethyl ether under unsaturated condition

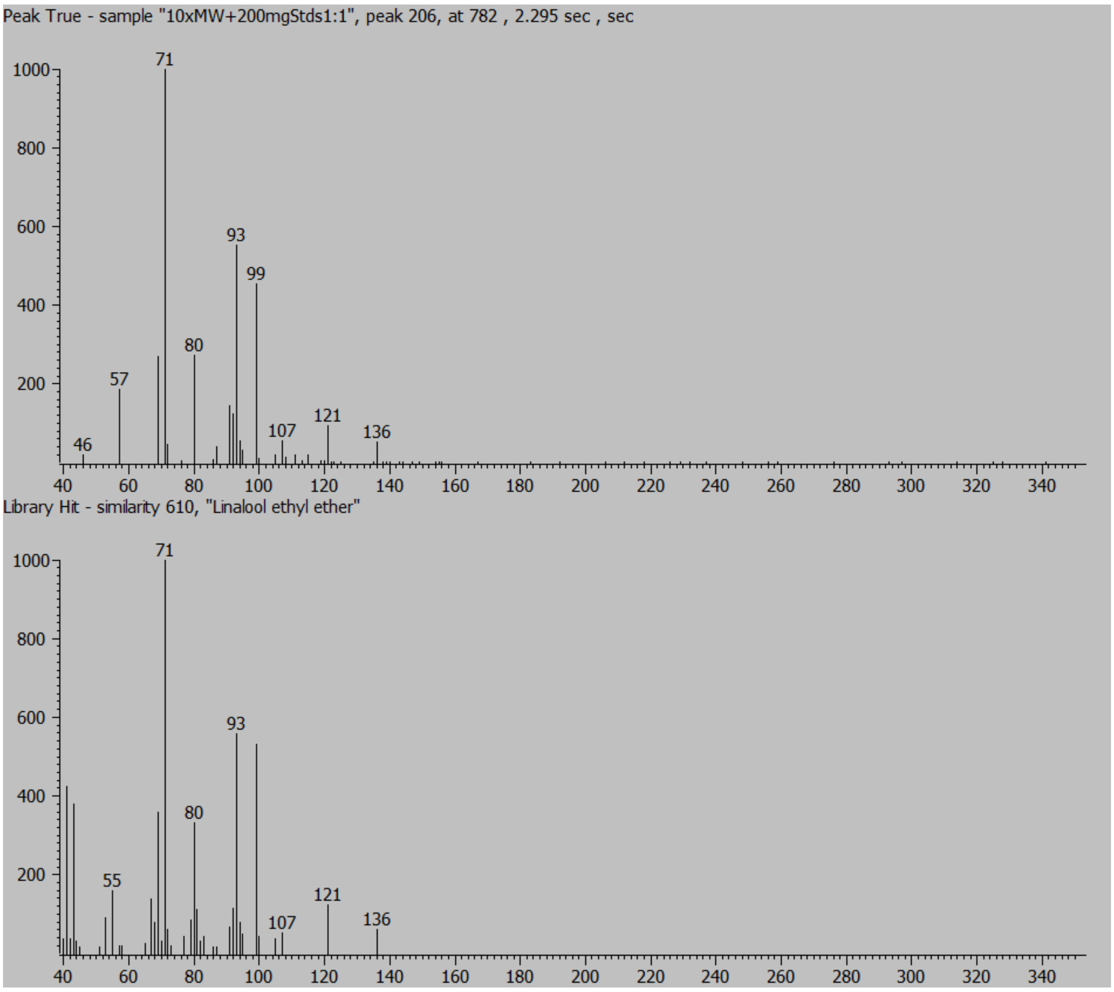

Figure S 8 the mass spectrum of linalool ethyl ether affected by saturation

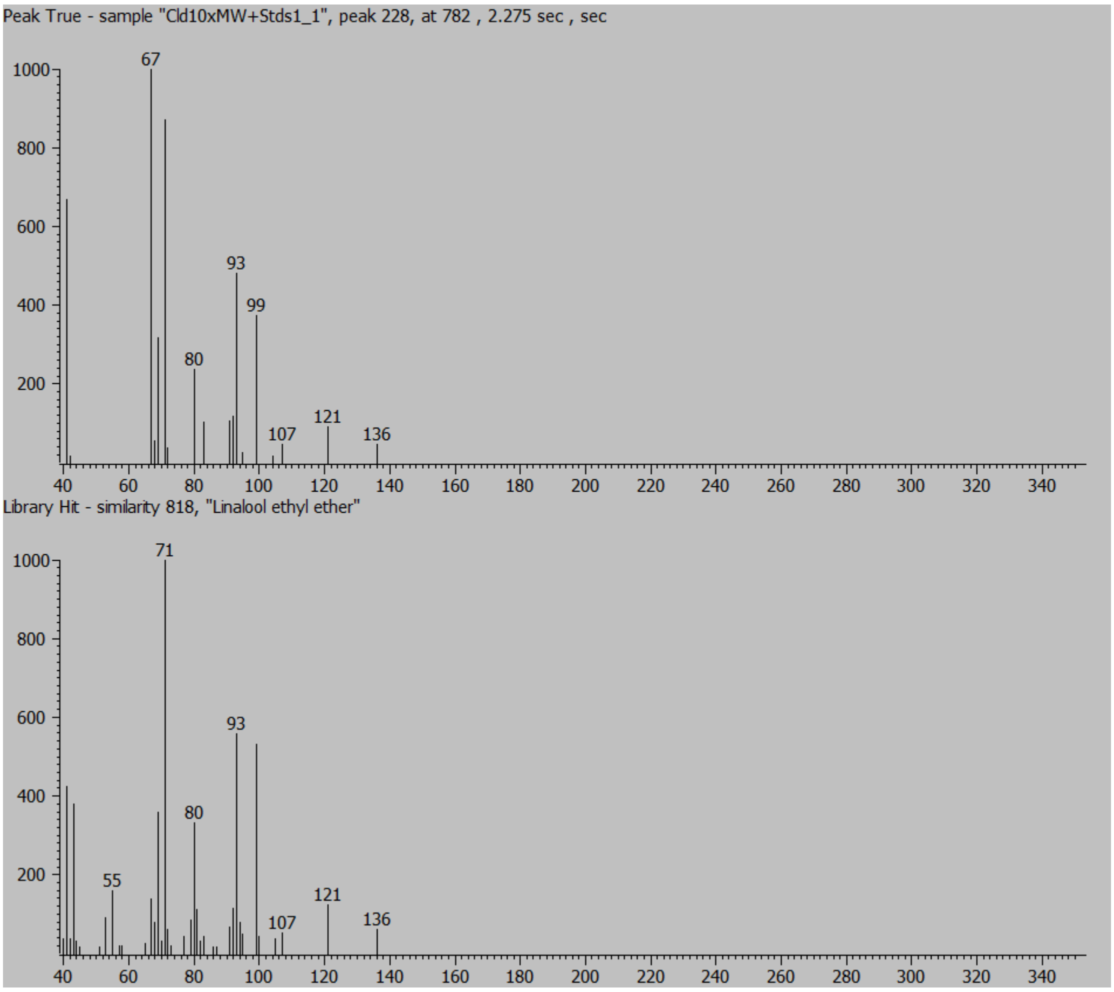

Figure S 9 the mass spectrum of linalool ethyl ether after saturation correction

Table S 2 summary information of the original peak table

	Total peak	Annotated peak
Diluted wine	546	400
Diluted wine + saturated standard	711	537
Saturated signal subtracted	1073	564
